# PP92 SWOT Analysis Of The AQuAS Health Technology Assessment Area’s Capacity To Generate Real-World Evidence Using Real-World Data

**DOI:** 10.1017/S0266462325103097

**Published:** 2025-12-29

**Authors:** Jessica Ruiz-Baena, Lucia Alonso García, Guillem Torres-Pagès, Clara Pons-Duran, Borja Velasco-Regúlez, Ramon Roman-Viñas, Rosa Maria Vivanco-Hidalgo

## Abstract

**Introduction:**

The use of real-world data (RWD) for real-world evidence (RWE) is essential for evidence-based decisions but requires robust technical, methodological, and organizational capabilities. We conducted an analysis of the Agència de Qualitat i Avaluació Sanitàries de Catalunya (AQuAS) Health Technology Assessment area to determine its capacity to generate RWE using RWD in Catalonia’s healthcare system, to identify priority areas to strengthen competencies and address emerging demands effectively.

**Methods:**

A structured SWOT (Strengths, Weaknesses, Opportunities, Threats) analysis was conducted, evaluating internal (strengths and weaknesses) and external (opportunities and threats) aspects. Data collection involved individual reflections and participatory workshops with the Assessment area’s team involved in RWE generation, focusing on critical areas such as data access, analytical capacities, technological resources, and regulatory environments. Finally, a strategic action plan was developed to implement appropriate measures.

**Results:**

Strengths: Consolidated expertise in data analysis and alliances with programs such as the Data Analytics Program for Research and Innovation in Health (PADRIS) in Catalonia; and availability of technological tools and procedures for RWE generation.

Weaknesses: Dependency on third parties for data access; centralized knowledge in key individuals; training gap in advanced methodologies (emulation of clinical trials and predictive analysis); and gaps in data homogenization and quality.

Opportunities: Potential to establish strategic alliances with other entities and leverage emerging technological advancements (implementing Observational Medical Outcomes Partnership standards).

Threats: Regulatory changes and competition in RWE development.

**Conclusions:**

SWOT analysis identified priority actions to enhance AQuAS’s capacity for RWE generation, including implementing training programs, formalizing strategic collaborations, and developing integrated processes for data management. This approach reinforced AQuAS’s role as a regional and international leader in using RWD for informed healthcare decision-making, highlighting the importance of capacity building and cooperation.

